# A nonsense mutation in *B3GALNT2* is concordant with hydrocephalus in Friesian horses

**DOI:** 10.1186/s12864-015-1936-z

**Published:** 2015-10-09

**Authors:** Bart J. Ducro, Anouk Schurink, John W. M. Bastiaansen, Iris J. M. Boegheim, Frank G. van Steenbeek, Manon Vos-Loohuis, Isaac J. Nijman, Glen R. Monroe, Ids Hellinga, Bert W. Dibbits, Willem Back, Peter A. J. Leegwater

**Affiliations:** Animal Breeding and Genomics Centre, Wageningen University, PO Box 338, 6700 AH Wageningen, The Netherlands; Department of Clinical Sciences of Companion Animals, Faculty of Veterinary Medicine, Utrecht University, PO Box 80154, 3508 TD Utrecht, The Netherlands; Department of Medical Genetics, University Medical Center Utrecht, PO Box 85090, 3508 AB Utrecht, The Netherlands; Koninklijke Vereniging “Het Friesch Paarden-Stamboek”, PO Box 624, 9200 AP Drachten, The Netherlands; Department of Equine Sciences, Faculty of Veterinary Medicine, Utrecht University, Yalelaan 112-114, 3584 CM Utrecht, The Netherlands; Department of Surgery and Anaesthesiology of Domestic Animals, Faculty of Veterinary Medicine, Ghent University, Salisburylaan 133, B-9820 Merelbeke, Belgium

**Keywords:** Hydrocephalus, *B3GALNT2*, Genome-wide association study, Next generation sequencing, Friesian horse, Muscular dystrophy

## Abstract

**Background:**

Hydrocephalus in Friesian horses is a developmental disorder that often results in stillbirth of affected foals and dystocia in dams. The occurrence is probably related to a founder effect and inbreeding in the population. The aim of our study was to find genomic associations, to investigate the mode of inheritance, to allow a DNA test for hydrocephalus in Friesian horses to be developed. In case of a monogenic inheritance we aimed to identify the causal mutation.

**Results:**

A genome-wide association study of hydrocephalus in 13 cases and 69 controls using 29,720 SNPs indicated the involvement of a region on ECA1 (*P* <1.68 × 10^−6^). Next generation DNA sequence analysis of 4 cases and 6 controls of gene exons within the region revealed a mutation in β-1,3-N-acetylgalactosaminyltransferase 2 (*B3GALNT2*) as the likely cause of hydrocephalus in Friesian horses. The nonsense mutation XM_001491545 c.1423C>T corresponding to XP_001491595 p.Gln475* was identical to a *B3GALNT2* mutation identified in a human case of muscular dystrophy-dystroglycanopathy with hydrocephalus. All 16 available cases and none of the controls were homozygous for the mutation, and all 17 obligate carriers (= dams of cases) were heterozygous. A random sample of the Friesian horse population (*n* = 865) was tested for the mutation in a commercial laboratory. One-hundred and forty-seven horses were carrier and 718 horses were homozygous for the normal allele; the estimated allele frequency in the Friesian horse population is 0.085.

**Conclusions:**

Hydrocephalus in Friesian horses has an autosomal recessive mode of inheritance. A nonsense mutation XM_001491545 c.1423C>T corresponding to XP_001491595 p.Gln475* in *B3GALNT2* (1:75,859,296–75,909,376) is concordant with hydrocephalus in Friesian horses. Application of a DNA test in the breeding programme will reduce the losses caused by hydrocephalus in the Friesian horse population.

**Electronic supplementary material:**

The online version of this article (doi:10.1186/s12864-015-1936-z) contains supplementary material, which is available to authorized users.

## Background

The Friesian horse breed originates from the Netherlands and is characterized by its specific appearance, gaits, versatility and character. Although the current size of the population is quite large, a small population size during certain periods in the past, combined with unequal genetic contribution of influential ancestors in a closed population, resulted in an inbreeding rate of 1.5 % per generation computed from pedigree data [1]. The computed inbreeding rate exceeds the FAO limit [2] of 1 % per generation, which is set to maintain the remaining genetic variation in a breed and by that limit the potential negative effects of inbreeding. Especially when caused by unequal genetic contribution, inbreeding can result in inbreeding depression and a higher incidence of genetic defects [3]. One such genetic defect that seems to occur predominantly in Friesian horses is congenital hydrocephalus [OMIA 000487-9796; [4–6]], although a few cases have been reported in other horse breeds like Standardbred trotters, Orlov trotters, a Finnish horse [7], American Miniature horses [8], Hanoverian warmblood horses [9] and Thoroughbred horses [10].

Hydrocephalus is defined as “an active distension of the ventricular system of the brain resulting from inadequate passage of cerebrospinal fluid (CSF) from its point of production within the cerebral ventricles to its point of absorption into the systemic circulation” [11]. Hydrocephalus can be acquired, e.g. due to infection or trauma, or can be hereditary in nature. To our best knowledge, no clear cases of a proven acquired hydrocephalus in horses have been reported in scientific literature. Different types of hydrocephalus have been identified based on the underlying mechanisms: communicating (increased production or impaired CSF absorption) or non-communicating (obstruction in CSF flow) [11]. Also, hydrocephalus can be internal or external, that is an accumulation of CSF respectively within or outside the ventricles of the brain. In horses, both external and internal hydrocephalus [7, 8] have been diagnosed. A Hannoverian foal affected with both internal and external hydrocephalus was reported as well [9]. Sipma et al. [6] examined 4 stillborn Friesian foals with hydrocephalus both macroscopically and microscopically and compared these affected foals with 2 unaffected stillborn Friesian foals. Noticeable on the outside of affected foals was the severe cranial distension. The most likely cause of hydrocephalus in Friesian horses, based on the findings from the examinations of these 4 foals, was an abnormal narrowing (stenosis) of the jugular foramen, suggesting “a communicative (external) hydrocephalus with a diminished absorption of cerebrospinal fluid into the systemic circulation at the venous sinuses due to a distorted, non-functional jugular foramen” [6]. Hydrocephalus also has been associated with dystocia in dams and can eventually lead to fatal complications for the dam at parturition [7, 8, 12]. Affected foals are often stillborn or are euthanized at birth to facilitate parturition. The incidence of hydrocephalus was 3.0 % in a population of 608 deformed fetuses or new-born foals, mostly Thoroughbreds, that were submitted for necropsy [13].

In humans, about 40 % of hydrocephalus cases have a known genetic cause according to the Online Mendelian Inheritance in Man (OMIM) database. Genetic heterogeneity of congenital hydrocephalus is observed: mutations in the *CCDC88C* gene [MIM:611204] on chromosome 14 [14, 15] and in the *MPDZ* gene [MIM:603785] on chromosome 9 [16] are responsible for autosomal recessive hydrocephalus (respectively HYC1 [MIM:236600] and HYC2 [MIM:615219]). The most common form of congenital hydrocephalus in humans (5 to 15 % of patients) is the X-linked recessive form caused by a mutation in *L1CAM* ([MIM:308840]; e.g. [17]; HSAS1 [MIM:307000]). Moreover, heterogeneity among phenotypes exists in humans as well as in animals [18]. To our best knowledge, research on the genetic background of hydrocephalus in horses is very limited. Ojala and Ala-Huikku [7] reported 10 foals with internal hydrocephalus of which 7 foals descended from the same Standardbred trotter stallion. This stallion was also the sire of a dam that aborted an affected foetus. A pedigree analysis was performed to determine the possible mode of inheritance (autosomal recessive, dominant or X-linked). The most likely cause of internal hydrocephalus in Standardbred trotters was a dominant mutation. The aim of our study was to find genomic associations, to investigate the mode of inheritance, to allow a DNA test for hydrocephalus in Friesian horses to be developed. In case of a monogenic inheritance we aimed to identify the causal mutation. Knowing the mutation will help in understanding the aetiology of hydrocephalus and allow selection against the disease allele using a DNA test. In our study, we identified a mutation concordant with hydrocephalus in Friesian horses using genotype and sequence data from affected and unaffected horses.

## Methods

### Phenotypes and horses

Phenotypic diagnosis of cases (*n* = 16) of hydrocephalus in Friesian horses was performed by local veterinarians. Controls (*n* = 89) were Friesian horses without the typical phenotypic appearance of hydrocephalus and included 17 dams of cases.

Cases and controls were born between 1995 and 2009. Stallions, geldings and mares were represented in the controls, while the gender of the cases was unknown. Paternal half-sib relationships were present between some of the cases, between some of the controls and also between some of the cases and controls. The pedigree information of several horses, however, turned out to be questionable, as large discrepancies were observed between pedigree relationship (**A**, according to Colleau [19]) and genomic relationship (**G**, according to VanRaden [20]) among horses calculated with calc_grm software [21] (data not shown). Relationships were not included in the analyses, but rather in the design phase of the project by choosing related controls for the cases.

### Samples, genotypes and quality control

Most of the control samples (*n* = 69) were obtained during treatment at the Equine Clinic of Utrecht University for reasons other than hydrocephalus. The remaining control samples were collected by the studbook during inspections, where paternal half-sibs of cases were sought. All samples were collected with permission of the horse’s owner (informed consent). Blood samples from controls were collected by jugular venepuncture in 10 ml blood collection tubes (Heparin or EDTA). DNA isolation from heparinized blood samples was performed as described by Orr et al. [22]. DNA isolation from blood samples collected in EDTA tubes was performed using the Gentra® Puregene® Kit from QIAGEN^©^ according to the manufacturer’s instructions. Samples from cases were voluntarily submitted by private owners through their local veterinarian to Utrecht University, which was facilitated by the studbook. DNA from cases was extracted from tissue samples (mostly ear, but also leg or spleen). Cases were patients of privately owned clinics and samples from controls were collected with informed consent of the horse’s owner. It was considered that there was no need for an Animal Care and Ethics Committee approval according to the Dutch law.

Genotypes of 13 cases and 69 controls (including 3 dams of cases) were obtained using the Illumina® EquineSNP50 Genotyping BeadChip containing 54,602 SNPs distributed evenly across the genome. SNPs with a minor allele frequency (MAF) <5 % and call-rate <90 % were discarded using the *check.marker* function in the GenABEL package in R [23]. Also, minimum call-rate per horse was set at 90 %. Almost all discarded SNPs (92.6 %) had MAF <5 %. In total 29,720 SNPs (54.4 % of all SNPs present on the BeadChip) were used in the analysis. Mean call-rate per horse after quality control was 99.6 %, and ranged from 91.3 to 99.97 %.

### Statistical analyses

The significance level of differences in genotype frequencies between cases and controls per SNP was determined with a *χ*^2^-test from 2 × 3 (genotypic) tables using the *ccfast* function in the GenABEL package in R [23]. The conservative Bonferroni corrected significance level was 1.68 × 10^−6^ ($$ =\raisebox{1ex}{$\alpha $}\!\left/ \!\raisebox{-1ex}{$n$}\right. $$, where *α* was the desired significance level being 0.05 and *n* was the number of SNPs (*n*≡ 29,720) that were tested).

Individual genotypes of cases and controls from all SNPs in the significantly associated region (60 to 90 Mb on ECA1) were visualized to allow an examination of the associated region to identify overlapping regions of homozygosity (identical-by-state) between cases.

### Next generation DNA sequence analysis

The procedures of the next generation sequencing were as described earlier [24, 25] and were performed by a designated laboratory of Utrecht University (Hubrecht Institute, Utrecht, the Netherlands). The exons and adjacent 20 bp of the genes in the region of interest were selected from the EquCab2.0 genome (Broad Institute, Cambridge, MA, USA) to design a Sure-Select slide (Agilent Technologies). The DNA of 4 randomly selected cases and 6 controls was sheared by sonication, tagged with individual DNA barcodes and pooled. These pools were size fractionated by gel electrophoresis. Fragments of 180–220 bp were excised and purified. The DNA was enriched for the selected exons by hybridization with the custom slide and sequenced using the SOLiD version 5500 system (Life Technologies).

DNA sequencing reads were mapped against the EquCab2.0 reference genome using BWA [26]. Only reads that were unambiguously mapped were used for variant calling by our custom caller. Briefly, variants were only considered if supported by ≥3 unique reads, when present at least 1× in the first 25 bases of a read (i.e. the region of a read that binds most accurately the target of interest, and has the highest sequencing quality), with a raw coverage ≥10×, base quality score ≥20 and a percentage of non-reference alleles ≥15 %.

### Sanger DNA sequencing

Due to its high GC content, exon 1 of *B3GALNT2* was not represented on the enrichment array and therefore not covered by the next generation sequencing experiment. For variant discovery, exon 1 was analysed by Sanger DNA sequencing in cases and controls. The PCR primer sequences were CAAGCCGACGTCAGCAGCAT and TTTCGTCTGGGGACCGTCGT. Amplification of exon 1 was performed using PCR-mix containing 1× Q5® Reaction Buffer, 1× Q5® High GC Enhancer, 1 M Betaine, dNTPs 0.2 mM each, 0.5 μM of each primer, 0.5 Unit Q5® Hot Start High-Fidelity DNA polymerase (New England Biolabs) and 50 ng of gDNA in a reaction volume of 15 μl. The thermal cycling protocol consisted of 30 s 98 °C followed by 40 cycles of 10 s at 98 °C, 30 s at 60 °C, 45 s at 72 °C, and a final elongation step at 72 °C for 2 min.

After identification of the candidate causal mutation, 16 available cases, 51 controls and 17 obligate carriers (dams of cases) were genotyped by Sanger sequencing for validation (Additional file 1). For this genotyping by sequencing step, PCR was performed with primers GTATGCATGCTCTCCGTCTT and AGTGCCAGGCCTAGAACTAT with 50 ng genomic DNA, 3 U Platinum Taq DNA polymerase (Invitrogen), 2 mM MgCl_2_, 0.2 mM each dNTP, 0.5 μM each primer and 1× Platinum buffer. Temperature cycling conditions were 5 min at 95 °C, 40 cycles of 30 s at 95 °C, 30 s at 55 °C, 30 s at 72 °C, and a final elongation step at 72 °C for 10 min. All amplifications were performed on an ABI 9700 Thermal Cycler (Applied Biosystems, Foster City, CA). DNA sequencing tercycle reactions were performed in the presence of 1 M Betaine using BigDye v3.1 (Applied Biosystems, Life Technologies) according to the manufacturer’s protocol. The products were analysed on a 3130XL Genetic Analyzer (Applied Biosystems, Life Technologies) and the data was analysed with Lasergene (version 11 DNASTAR).

### Population screening

A DNA test for the candidate causal mutation has been developed to be able to genotype broodmares and stallions and is now available at a commercial laboratory. Until now, 805 mares and 60 stallions have been tested.

### Linkage disequilibrium

Linkage disequilibrium (LD, as squared correlation coefficient *r*^2^ [27]) between SNPs and between SNPs and the mutation was calculated using Haploview 4.2 [28].

## Results

### Associated SNPs and homozygosity

**Fig. 1:**
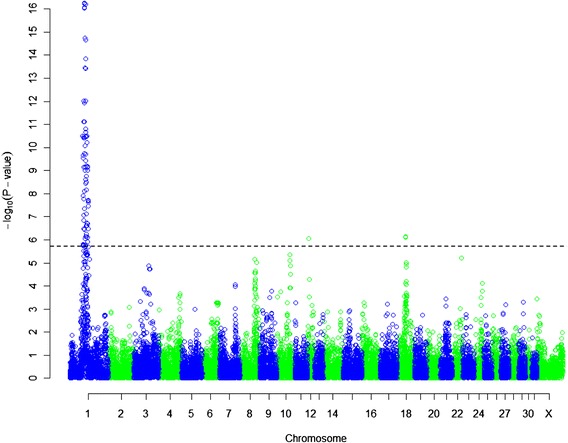
Manhattan plot of hydrocephalus in Friesian horses. Significance level based on genotype differences between cases (*n* = 13) and controls (*n* = 69) using a *χ* ^2^-test (2df). The *horizontal line* is the Bonferroni corrected significance level (*P* = 1.68 × 10^−6^)

The genome-wide association study implicated a region on *Equus caballus* autosome (ECA) 1 to be associated with hydrocephalus. Of the 99 SNPs that passed the Bonferroni corrected significance level, most (*n* = 96) were located on ECA1 (Fig. 1). SNPs significantly associated with hydrocephalus in Friesian horses on ECA1 were located between 61 and 87 Mb (Additional file 2). Six SNPs (BIEC2-28302, BIEC2-28459, BIEC2-28506, BIEC2-28912, BIEC2-28931 and BIEC2-28955) were most strongly associated (*P* = 5.64 × 10^−17^) with hydrocephalus in Friesian horses (Additional file 2). Genotype distribution of these 6 SNPs, as well as for almost all SNPs significantly associated with hydrocephalus, were consistent with an autosomal recessive mode of inheritance of hydrocephalus (Additional file 2).

**Fig. 2:**
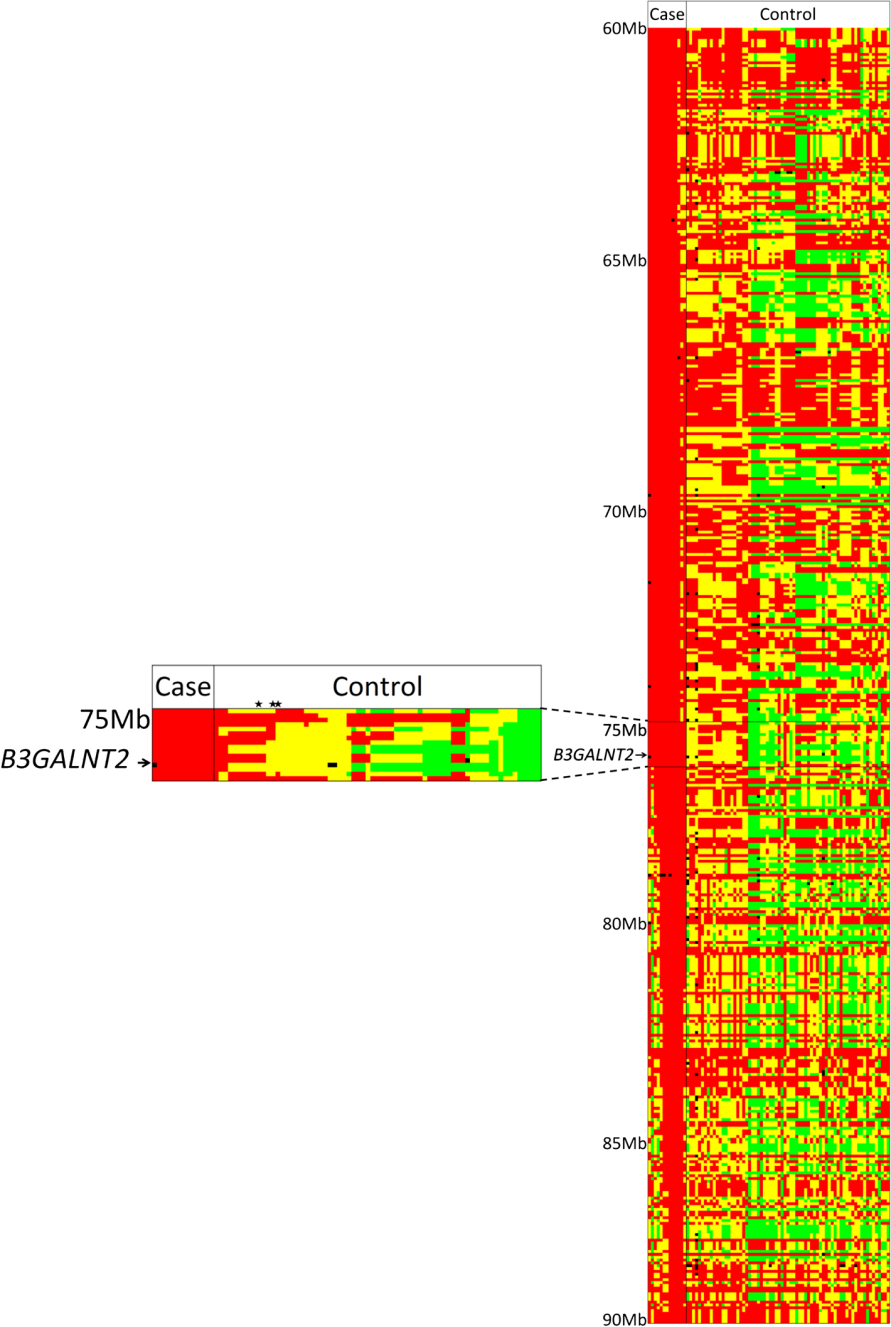
SNP genotypes from 60 to 90 Mb on ECA1 for cases (*n* = 13) and controls (*n* = 69). Hydrocephalus associated homozygous genotypes are depicted by *red*, heterozygous genotypes by *yellow*, normal homozygous genotypes by *green* and missing genotypes by *black*. Each *column* corresponds to one horse, each *row* corresponds to one SNP. The candidate mutation in *B3GALNT2* is part of a region of 1.47 Mb in length (range: 74,897,451–76,370,694) that is shared homozygous by all 13 cases and one control. The region of 1.47 Mb in length is shown in the enlargement, where obligate carriers (dams of cases) are marked with a *star*

A display of SNP genotypes from 60 to 90 Mb on ECA1 showed that homozygosity extended for 10.5 Mb (from 65.9 to 76.4 Mb [29]) in 12 out of 13 cases (Fig. 2). There were several overlapping regions of homozygosity between all 13 cases (Fig. 2). One region displayed homozygosity in all 13 cases and only one control. This region was located at position 74,897,451–76,370,694 (1.47 Mb) and included 18 genes (Additional file 3). At the point in time that we reached this stage, none of the genes was a clear candidate for the hydrocephalus phenotype observed in the Friesian horses.

### DNA sequence analysis

A broad region surrounding the shared region of homozygosity was analysed by sequencing the coding exons and exon-intron junctions of the genes in the region between position 59.6 and 78.0 Mb of ECA1 identified on the *Equus caballus* reference genome assembly EquCab2.0, annotation release 101. The region contained 142 genes with 1453 coding exons (Additional file 4). Two of the exons were skipped due to design problems. A broad region was selected to circumvent the risk of mapping artefacts because of phenocopies in the group of cases. DNA sequence reads were generated of 4 cases and 6 controls. Of the reads, on average 60 % was derived from the targeted region. The average coverage of the region was 216 fold per DNA sample, and ranged from 124 fold to 332 fold. Of the variable positions found in the regions shared in a homozygous state by the 13 cases that were included in the genome-wide analysis, 3 fitted the case–control model for a recessive disorder (Additional file 5). Two of these were known variations in the genes *LYST* and *NID1* (rs394520771 and rs394402493, respectively). The variant in the gene coding for β-1,3-N-acetylgalactosaminyltransferase 2 (*B3GALNT2* [Ensembl:ENSECAG00000013338]) was a very likely candidate for the hydrocephalus phenotype. The mutation introduced a stopcodon on ECA1:75,907,505 (exon 12), c.1423C>T [GenBank:XM_001491545] corresponding to p.Gln475* [GenBank:XP_001491595]. In humans, a muscular dystrophy-dystroglycanopathy phenotype [MDDGA11, MIM:615181] including hydrocephalus has recently been associated with mutations in *B3GALNT2* [30, 31]. The 4 affected Friesian horses were homozygous for the nonsense allele, while one of the controls was heterozygous and the others were homozygous for the normal allele.

**Fig. 3:**
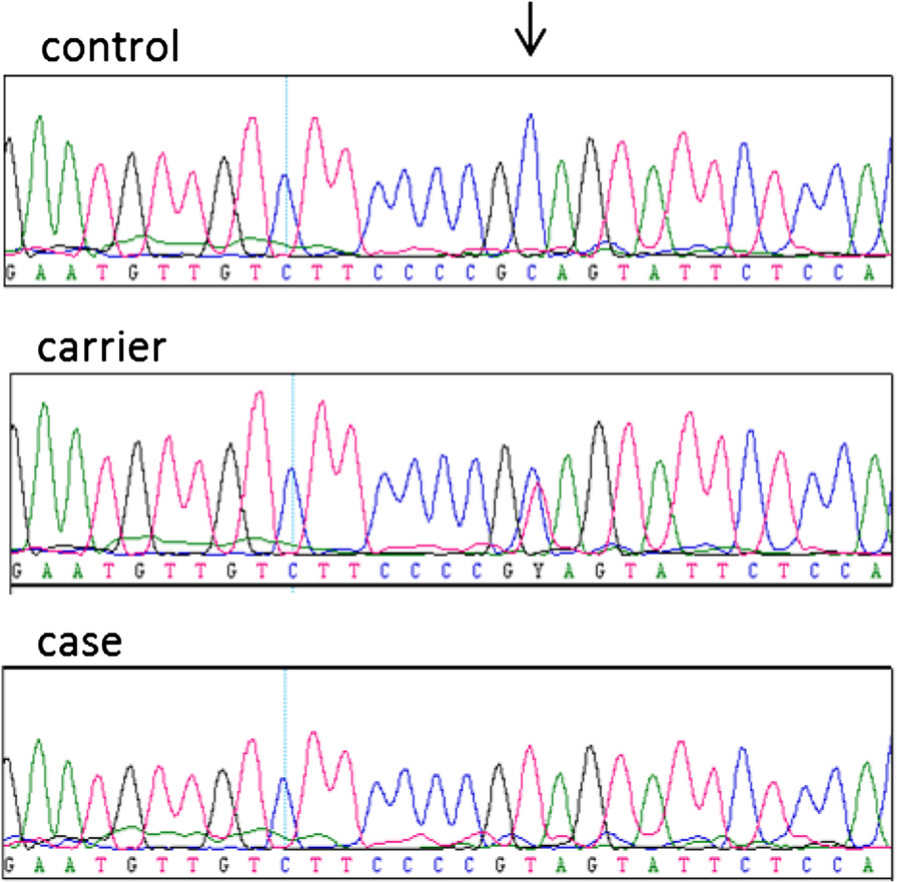
DNA sequencing results of a case, obligate carrier and control. The mutation in the gene *B3GALNT2* that causes hydrocephalus in Friesian horses. Fragments comprising exon 12 of the gene from an affected foetus (= case), an obligate carrier and a control horse were amplified by PCR and analysed by Sanger DNA sequencing. The C>T mutation (*arrow*) changes the codon CAG for glutamine into a premature TAG stop codon, truncating the encoded protein by 26 amino acids. The nomenclature for the mutation is c.1423C>T [GenBank:XM_001491545] corresponding to p.Gln475* [GenBank:XP_001491595]

The 16 cases of Friesian horses with hydrocephalus that were available for the validation were genotyped for the mutation by Sanger DNA sequencing and all were found homozygous (TT) (Additional file 1). None of the healthy controls was homozygous for the mutation. There were 32 heterozygotes (TC), including all 17 obligate carriers, and 36 horses were homozygous for the normal allele (CC) (Fig. 3). These numbers do not reflect the allele frequency in the population because the horses were not randomly selected. Healthy controls descending from carrier sires were overrepresented. No variations were observed by Sanger DNA sequencing of exon 1 from *B3GALNT2*, which was not covered by the next generation sequencing experiment.

### Population screening

Out of 60 stallions that were genotyped using a commercially available DNA test based on the *B3GALNT2* mutation, 8 (13.3 %) were carrier of the allele T. Out of 805 broodmares, 139 (17.3 %) were also carrier. As the tested horses can be considered as a random sample of the population, the estimated frequency of allele T in the Friesian horse population is 0.085( = (8 + 139) / ((60 + 805) × 2)). None of the tested (unaffected) horses were homozygous for the mutation (TT).

### Linkage disequilibrium

**Fig. 4:**
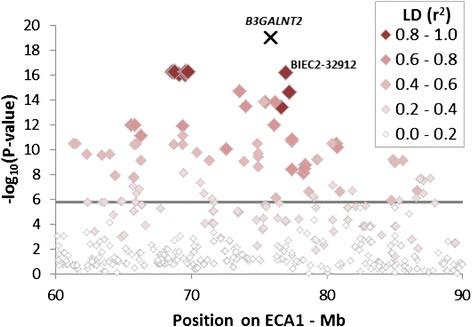
Regional association plot of the region (on ECA1) most significantly associated with hydrocephalus in Friesian horses. Significance level based on genotype differences between cases (*n* = 13) and controls (*n* = 69) using a *χ* ^2^-test (2df). The *horizontal line* is the genome-wide Bonferroni corrected significance level (*P* = 1.68 × 10^−6^). Location of the *B3GALNT2* gene [Ensembl:ENSECAG00000013338] is indicated by the *black cross*. SNPs surrounding *B3GALNT2* are coloured in diminishing *red* to reflect LD (pair-wise *r* ^2^ values) between the candidate mutation in *B3GALNT2* and SNPs. LD calculations between the mutation and the surrounding SNPs are based on genotypes for the mutation of 13 cases, 45 controls and 3 dams of cases and on SNP genotypes of all 13 cases and 69 controls

LD between the candidate mutation in *B3GALNT2* and surrounding SNPs was highest (0.929) for XM_001491545 c.1423C>T and BIEC2-32912 (Additional file 6). This SNP is located 1.1 Mb away from *B3GALNT2* and was significantly associated with hydrocephalus (*P* = 6.13 × 10^−17^; Fig. 4). LD was 0.928 between the mutation and the SNPs most significantly associated with hydrocephalus (*P* = 5.64 × 10^−17^). However, the distance between XM_001491545 c.1423C>T and the closest of these SNPs was 6.1 Mb (Fig. 4).

## Discussion

**Fig. 5:**
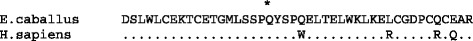
Conservation of the C-terminal end of the B3GALNT2 protein between horse and man. The depicted 44 amino acid sequence is encoded by exon 12 of the gene in the horse. Identical amino acids in man are indicated by a *dot*. The point of truncation in Friesian horses with hydrocephalus and a human patient with muscular dystrophy-dystroglycanopathy type A, 11 [30] is indicated by an *asterisk*. E.caballus = horse, H.sapiens = human

Hydrocephalus has been recognized in many species, in both livestock, laboratory animals and pets [32–36]. The autosomal recessive mutation responsible for a neuropathic form of hydrocephalus has been identified in Angus beef cattle (OMIA:000487-9913, Hydrocephalus in *Bos taurus*). In mice, an autosomal recessive mutation in *HYDIN* (hydrocephalus-inducing [Ensembl:ENSMUSG00000059854]) causes a lethal form of a communicating hydrocephalus with a perinatal onset. A 2-bp deletion in exon 15 results in a premature termination of the protein [37]. The mode of inheritance of hydrocephalus in Friesian horses appears to be autosomal recessive and genetic heterogeneity was not observed within our sample of the population. In humans, however, genetic heterogeneity of congenital hydrocephalus has been observed. Mutations in several genes, both X-linked and autosomal, are responsible for hydrocephalus. In the current study, no genetic heterogeneity was observed for hydrocephalus with all 16 affected Friesian horses being homozygous for the same nonsense mutation in *B3GALNT2*. The strongest indication that this mutation causes the phenotype in Friesian horses is that the exact same nonsense mutation was found homozygously in a human muscular dystrophy patient with hydrocephalus by Stevens et al. [30] (Fig. 5) [MIM:610194.0006 or dbSNP:rs367543077]. Nine different mutations were observed in the *B3GALNT2* gene in 6 human patients with muscular dystrophy-dystroglycanopathy with brain and eye anomalies, type A, 11 (MDDGA11 [MIM:615181]). Following Stevens et al., Hedberg et al. [31] identified 2 novel mutations in *B3GALNT2* in a 5-year-old girl with milder clinical features (ataxia, spasticity, muscle weakness) and brain abnormalities (e.g. cerebellar cysts) detected using MRI. The syndrome was clinically somewhat variable and only 4 patients and 1 affected foetus (from 3 families) displayed hydrocephalus [30, 31]. Searches of OMIM, OMIA, Ensembl and NCBI databases have not revealed associations between *B3GALNT2* and a phenotype or disorder other than muscular dystrophy-dystroglycanopathy in humans.

Two other missense variants were observed by next generation sequencing that segregated with the hydrocephalus phenotype. Both these variations were annotated in dbSNP by two laboratories and were observed in Quarter horses and an undefined sample of horses. One variation was located in *LYST* (rs394520771). Mutations in this gene cause Chediak-Higashi syndrome (MIM:214500) in humans, of which hydrocephalus is not a characteristic. The other variation was located in *NID1* (rs394402493). Mice with knock-out mutations of this gene do not display a phenotype.

A subset of congenital muscular dystrophies in humans (including MDDGA11), called dystroglycanopathies, is characterized by a reduced glycosylation of α-dystroglycan (α-DG), which is an integral component of the dystrophin glycoprotein complex. Dystroglycan is present in skeletal muscle but also in many tissues like the brain, where it has a multitude of functions such as morphogenesis and early development [38]. Dystroglycanopathies are clinically heterogeneous disorders and are often associated with central nervous system pathology [39]. Mutations in several genes have been associated with dystroglycanopathies, although still in about half of the patients with dystroglycanopathy no mutation was found [40]. Results from Stevens et al. [30] and Hedberg et al. [31] demonstrated that *B3GALNT2* is involved in the glycosylation of α-DG and that mutations within this gene can cause muscular dystrophy-dystroglycanopathy with muscle and brain anomalies. In Friesian horses, muscular dystrophy was never considered, because affected foals are often stillborn and dystrophy was therefore not observed clinically. Based on the findings from the macroscopic and microscopic examination of 4 stillborn Friesian foals with hydrocephalus, the most likely cause of hydrocephalus in Friesian horses was considered to be an abnormal narrowing (stenosis) of the jugular foramen [6]. Because the exact same nonsense mutation of *B3GALNT2* found in Friesian horses was also present in a MDDGA11 human patient with hydrocephalus, the same complex as in humans (including muscular dystrophy) might underlie the phenotype observed in Friesian horses. A subsequent immunohistochemical examination of muscle biopsies from hydrocephalus cases might determine whether these cases suffer from muscular dystrophy as well.

The genome-wide association study identified a region on ECA1 highly associated with hydrocephalus. Due to the autosomal recessive mode of inheritance for hydrocephalus in Friesian horses, the 2 × 3 tables to test differences in genotype frequencies between cases and controls for most of the significantly associated SNPs contained cell(s) with zero observations. Therefore, the Fisher’s exact test would have been a more suitable test to use compared to the applied *χ*^2^-test. Although the calculated significance level between these two tests could differ, the same region on ECA1 was still found to be strongly associated with hydrocephalus when applying the Fisher’s exact test. We illustrated that indeed the identification of a region associated with a phenotype in a population with reasonable inbreeding (and LD) is feasible. However, fine-mapping was hampered. Within such populations, a sufficiently large region surrounding the significantly associated SNPs should be sequenced to be able to identify the causal mutation. Most of the highly significantly associated SNPs (except BIEC2-32912) were located more than 6 Mb away from *B3GALNT2* (ECA1:75,859,296-75,909,376). Also, the homozygosity in 12 out of 13 cases extended over several mega base pairs (≥10.5 Mb; Fig. 2). There were several overlapping regions of homozygosity between all 13 cases (Fig. 2), but these regions were shared in a homozygous state by 4 or more controls. However, there was one region of 1.47 Mb in length (from 74,897,451 to 76,370,694 bp, *Equus caballus* EquCab2.0 reference genome [29], Fig. 2), where all 13 cases and only one of the controls were homozygous for the hydrocephalus associated allele that was defined by 16 consecutive SNPs. The candidate gene *B3GALNT2* is located within this region of 1.47 Mb and the genotype of the one homozygous control at the presumed causal mutation was TC. Homozygosity mapping indicated a recombination event close to the mutation in *B3GALNT2*, as one out of 13 cases did not show the entire 10.5 Mb region as identical-by-state. Therefore, hydrocephalus in Friesian horses presumably is caused by a recent mutation on ECA1 that occurred in this breed.

The subsequent genotyping of Friesian horses for the mutation will identify carriers in unaffected horses. Exclusion of carriers from breeding will be the fastest way to diminish hydrocephalus incidence. However, exclusion of all carriers will likely not be a sound strategy for this population as inbreeding is reasonable [1]. Inbreeding can increase faster when carriers are excluded. For now, breeders are informed about the mutation status (carrier/non-carrier) of newly approved young stallions and can voluntarily test their own mares. Mating 2 carriers should be avoided. Other measures, such as testing of embryos before implantation may be feasible, but this is currently not commonly practiced yet in Friesian horses.

## Conclusions

A nonsense mutation in *B3GALNT2* is concordant with hydrocephalus in Friesian horses, which has an autosomal recessive mode of inheritance. A genome-wide association study and homozygosity mapping identified a region on ECA1 highly associated with hydrocephalus. Subsequent analysis of sequence data identified a putative causal mutation, which was successfully validated in both affected and unaffected Friesian horses. Carriers among unaffected horses can be identified and the application of this knowledge in the breeding programme will thus reduce the losses caused by hydrocephalus in the Friesian horse population.

## Additional files

Additional file 1:
**Sanger DNA sequencing results for the nonsense mutation.** A table with information on all horses that have been sequenced for the nonsense mutation. (DOCX 20 kb) Additional file 2:
**SNPs significantly associated with hydrocephalus in Friesian horses.** A table with information on all 99 SNPs that passed the Bonferroni corrected significance level. (DOCX 41 kb) Additional file 3:
**Genes located in the ECA1 region shared in a homozygous state by hydrocephalus cases.** Start and stop position (in base pair; *Equus caballus* EquCab2.0 reference genome [29]), symbol and description of genes located in the region of 1.47 Mb in length that is shared in a homozygous state by hydrocephalus cases. (DOCX 21 kb) Additional file 4:
**Coding exons selected for Next Generation Sequencing in Friesian horses with hydrocephalus.** A .bed file describing the targeted regions, including chromosome number, start and end position (in base pair; *Equus caballus* EquCab2.0 reference genome [29]). (TXT 34 kb) Additional file 5:
**DNA sequence variations in regions of homozygosity in Friesian horses with hydrocephalus.** A table with information on DNA sequence variations in regions of homozygosity in Friesian horses with hydrocephalus. (DOCX 20 kb) Additional file 6:
**Linkage disequilibrium between the candidate mutation in** ***B3GALNT2*** **and surrounding SNPs.** A figure with LD between the candidate mutation in *B3GALNT2* and surrounding SNPs. (DOCX 174 kb)

## Abbreviations

ECA: *Equus caballus*
CSF: Cerebrospinal fluid
OMIM: Online Mendelian Inheritance in Man
OMIA: Online Mendelian Inheritance in Animals
B3GALNT2: β-1,3-N-acetylgalactosaminyltransferase 2
