# Supplementary material for: A nonsense mutation in B3GALNT2 is concordant with hydrocephalus in Friesian horses
Source: BMC Genomics. 2015 Oct 9;16:761. doi: 10.1186/s12864-015-1936-z (PMC4600337; doi:10.1186/s12864-015-1936-z)
Supplement: Additional file 1: — Sanger DNA sequencing results for the nonsense mutation. A table with information on all horses that have been sequenced for the nonsense mutation. (DOCX 20 kb) [file 12864_2015_1936_MOESM1_ESM.docx]

## Sanger DNA sequencing results for the nonsense mutation

Genotype for the nonsense mutation for cases and controls, including information on whether the horse has been genotyped using the Illumina^®^ EquineSNP50 Genotyping BeadChip (50K), sequenced using a next-generation sequencing procedure (NGS) or sequenced using Sanger sequencing (Sanger). 21 controls that were included in the genome-wide association study were not sequenced for the nonsense mutation.

| Phenotype | Genotype | Number | 50K | NGS | Sanger |
| --- | --- | --- | --- | --- | --- |
| Case | TT | 3 | Yes | Yes | Yes |
| Case | TT | 10 | Yes | No | Yes |
| Case | TT | 1 | No | Yes | Yes |
| Case | TT | 2 | No | No | Yes |
| Control | TC | 1 | Yes | Yes | Yes |
| Control | TC | 13 | Yes | No | Yes |
| Control (dam of case) | TC | 3 | Yes | No | Yes |
| Control (dam of case) | TC | 14 | No | No | Yes |
| Control | TC | 1 | No | No | Yes |
| Control | CC | 5 | Yes | Yes | Yes |
| Control | CC | 26 | Yes | No | Yes |
| Control | CC | 5 | No | No | Yes |
| Control | - | 21 | Yes | No | No |
