# Supplementary material for: A nonsense mutation in B3GALNT2 is concordant with hydrocephalus in Friesian horses
Source: BMC Genomics. 2015 Oct 9;16:761. doi: 10.1186/s12864-015-1936-z (PMC4600337; doi:10.1186/s12864-015-1936-z)
Supplement: Additional file 2: — SNPs significantly associated with hydrocephalus in Friesian horses. A table with information on all 99 SNPs that passed the Bonferroni corrected significance level. (DOCX 41 kb) [file 12864_2015_1936_MOESM2_ESM.docx]

## SNPs significantly associated with hydrocephalus in Friesian horses

99 SNPs passed the Bonferroni corrected significance level (= 1.68 × 10^-6^). Chromosome (ECA = *Equus caballus*), position (in base pair; *Equus caballus* EquCab2.0 reference genome [29]), P-value (χ^2^-test with 2df) and frequency of the hydrocephalus associated homozygous genotype (AA), heterozygous genotype (AB) and homozygous genotype (BB) in cases (n = 13) and controls (n = 69) are presented per SNP. Location of *B3GALNT2* (1:75,859,296-75,909,376) is between BIEC2-32205 and BIEC2-32505. The grey shaded SNPs are the SNPs most significantly associated with hydrocephalus and in complete LD with each other.

|  |  |  |  |  | Frequency genotype | | | | | | |
| --- | --- | --- | --- | --- | --- | --- | --- | --- | --- | --- | --- |
|  |  |  |  |  | Cases | | |  | Controls | | |
| SNP | ECA | Position | P-value |  | AA | AB | BB |  | AA | AB | BB |
| BIEC2-25335 | 1 | 61,311,028 | 3.25 × 10^-11^ |  | 12 | 1 | 0 |  | 5 | 29 | 35 |
| BIEC2-25357 | 1 | 61,479,005 | 3.25 × 10^-11^ |  | 12 | 1 | 0 |  | 5 | 29 | 35 |
| BIEC2-25551 | 1 | 62,260,532 | 2.21 × 10^-10^ |  | 10 | 3 | 0 |  | 3 | 27 | 39 |
| BIEC2-26016 | 1 | 63,415,184 | 2.92 × 10^-10^ |  | 10 | 3 | 0 |  | 3 | 26 | 39 |
| BIEC2-26386 | 1 | 63,975,987 | 2.21 × 10^-10^ |  | 10 | 3 | 0 |  | 3 | 27 | 39 |
| BIEC2-26590 | 1 | 64,395,060 | 1.21 × 10^-08^ |  | 10 | 3 | 0 |  | 5 | 32 | 32 |
| BIEC2-26699 | 1 | 64,771,094 | 1.58 × 10^-06^ |  | 11 | 2 | 0 |  | 11 | 30 | 28 |
| BIEC2-26736 | 1 | 64,910,330 | 1.53 × 10^-06^ |  | 11 | 2 | 0 |  | 11 | 28 | 30 |
| BIEC2-27143 | 1 | 65,553,951 | 9.54 × 10^-13^ |  | 11 | 2 | 0 |  | 2 | 27 | 40 |
| BIEC2-27258 | 1 | 65,709,486 | 8.58 × 10^-08^ |  | 11 | 2 | 0 |  | 8 | 37 | 23 |
| BIEC2-27280 | 1 | 65,732,134 | 1.66 × 10^-08^ |  | 11 | 2 | 0 |  | 7 | 32 | 30 |
| BIEC2-27301 | 1 | 65,769,727 | 1.66 × 10^-08^ |  | 11 | 2 | 0 |  | 7 | 32 | 30 |
| BIEC2-27336 | 1 | 65,814,777 | 9.54 × 10^-13^ |  | 11 | 2 | 0 |  | 2 | 27 | 40 |
| BIEC2-27351 | 1 | 65,841,804 | 3.98 × 10^-11^ |  | 13 | 0 | 0 |  | 7 | 33 | 29 |
| BIEC2-27352 | 1 | 65,842,030 | 3.98 × 10^-11^ |  | 13 | 0 | 0 |  | 7 | 33 | 29 |
| BIEC2-27377 | 1 | 65,869,896 | 1.64 × 10^-06^ |  | 11 | 2 | 0 |  | 11 | 33 | 25 |
| BIEC2-27378 | 1 | 65,869,949 | 1.64 × 10^-06^ |  | 11 | 2 | 0 |  | 11 | 33 | 25 |
| BIEC2-27399 | 1 | 65,922,084 | 3.31 × 10^-11^ |  | 12 | 1 | 0 |  | 5 | 34 | 30 |
| BIEC2-27405 | 1 | 65,933,897 | 3.39 × 10^-07^ |  | 13 | 0 | 0 |  | 15 | 33 | 21 |
| BIEC2-27499 | 1 | 66,142,552 | 1.47 × 10^-07^ |  | 13 | 0 | 0 |  | 14 | 33 | 22 |
| BIEC2-27588 | 1 | 66,296,523 | 7.52 × 10^-12^ |  | 13 | 0 | 0 |  | 6 | 28 | 35 |
| BIEC2-27590 | 1 | 66,302,733 | 7.09 × 10^-10^ |  | 13 | 0 | 0 |  | 9 | 33 | 27 |
| BIEC2-28302 | 1 | 68,597,487 | 5.64 × 10^-17^ |  | 12 | 1 | 0 |  | 0 | 22 | 47 |
| BIEC2-28459 | 1 | 68,701,400 | 5.64 × 10^-17^ |  | 12 | 1 | 0 |  | 0 | 22 | 47 |
| BIEC2-28464 | 1 | 68,708,147 | 3.98 × 10^-11^ |  | 13 | 0 | 0 |  | 7 | 36 | 26 |
| BIEC2-28506 | 1 | 68,778,089 | 5.64 × 10^-17^ |  | 12 | 1 | 0 |  | 0 | 22 | 47 |
| BIEC2-28507 | 1 | 68,778,169 | 5.71 × 10^-17^ |  | 12 | 1 | 0 |  | 0 | 24 | 45 |
| BIEC2-28749 | 1 | 69,062,965 | 8.97 × 10^-17^ |  | 12 | 1 | 0 |  | 0 | 22 | 46 |
| BIEC2-28874 | 1 | 69,326,275 | 7.52 × 10^-12^ |  | 13 | 0 | 0 |  | 6 | 34 | 29 |
| BIEC2-28875 | 1 | 69,339,109 | 1.18 × 10^-12^ |  | 13 | 0 | 0 |  | 5 | 29 | 35 |
| BIEC2-28876 | 1 | 69,339,208 | 3.98 × 10^-11^ |  | 13 | 0 | 0 |  | 7 | 34 | 28 |
| BIEC2-28888 | 1 | 69,407,277 | 7.81 × 10^-09^ |  | 13 | 0 | 0 |  | 11 | 40 | 18 |
| BIEC2-28892 | 1 | 69,407,512 | 7.81 × 10^-09^ |  | 13 | 0 | 0 |  | 11 | 40 | 18 |
| BIEC2-28898 | 1 | 69,410,844 | 7.81 × 10^-09^ |  | 13 | 0 | 0 |  | 11 | 40 | 18 |
| BIEC2-28907 | 1 | 69,505,589 | 9.08 × 10^-17^ |  | 12 | 1 | 0 |  | 0 | 24 | 44 |
| BIEC2-28908 | 1 | 69,543,750 | 8.90 × 10^-17^ |  | 12 | 1 | 0 |  | 0 | 21 | 47 |
| BIEC2-28912 | 1 | 69,561,176 | 5.64 × 10^-17^ |  | 12 | 1 | 0 |  | 0 | 22 | 47 |
| BIEC2-28931 | 1 | 69,697,043 | 5.64 × 10^-17^ |  | 12 | 1 | 0 |  | 0 | 22 | 47 |
| BIEC2-28955 | 1 | 69,736,571 | 5.64 × 10^-17^ |  | 12 | 1 | 0 |  | 0 | 22 | 47 |
| BIEC2-29359 | 1 | 70,378,596 | 1.80 × 10^-10^ |  | 13 | 0 | 0 |  | 8 | 33 | 28 |
| BIEC2-29471 | 1 | 70,661,243 | 4.54 × 10^-08^ |  | 12 | 1 | 0 |  | 10 | 37 | 22 |
| BIEC2-29595 | 1 | 70,953,234 | 3.48 × 10^-07^ |  | 12 | 1 | 0 |  | 12 | 34 | 23 |
| BIEC2-29857 | 1 | 71,344,522 | 7.09 × 10^-10^ |  | 13 | 0 | 0 |  | 9 | 37 | 23 |
| BIEC2-29865 | 1 | 71,348,106 | 7.09 × 10^-10^ |  | 13 | 0 | 0 |  | 9 | 37 | 23 |
| BIEC2-30016 | 1 | 71,495,554 | 8.97 × 10^-07^ |  | 11 | 1 | 0 |  | 12 | 31 | 26 |
| BIEC2-30716 | 1 | 72,537,262 | 8.25 × 10^-11^ |  | 12 | 1 | 0 |  | 5 | 18 | 43 |
| BIEC2-31300 | 1 | 73,545,254 | 1.85 × 10^-15^ |  | 12 | 1 | 0 |  | 1 | 28 | 40 |
| BIEC2-31328 | 1 | 73,575,357 | 1.85 × 10^-15^ |  | 12 | 1 | 0 |  | 1 | 28 | 40 |
| BIEC2-31514 | 1 | 73,881,378 | 9.60 × 10^-10^ |  | 13 | 0 | 0 |  | 9 | 35 | 24 |
| BIEC2-31580 | 1 | 73,992,006 | 3.52 × 10^-14^ |  | 12 | 1 | 0 |  | 2 | 24 | 43 |
| BIEC2-32009 | 1 | 74,880,142 | 3.37 × 10^-11^ |  | 12 | 1 | 0 |  | 5 | 40 | 24 |
| BIEC2-32016 | 1 | 74,897,083 | 1.95 × 10^-10^ |  | 12 | 1 | 0 |  | 6 | 37 | 26 |
| BIEC2-32021 | 1 | 74,897,451 | 3.65 × 10^-10^ |  | 12 | 1 | 0 |  | 6 | 35 | 26 |
| BIEC2-32043 | 1 | 74,938,827 | 2.48 × 10^-09^ |  | 13 | 0 | 0 |  | 10 | 37 | 22 |
| BIEC2-32168 | 1 | 75,308,260 | 1.45 × 10^-14^ |  | 13 | 0 | 0 |  | 3 | 41 | 25 |
| BIEC2-32202 | 1 | 75,458,447 | 1.45 × 10^-14^ |  | 13 | 0 | 0 |  | 3 | 28 | 38 |
| BIEC2-32205 | 1 | 75,461,775 | 1.45 × 10^-14^ |  | 13 | 0 | 0 |  | 3 | 28 | 38 |
| BIEC2-32505 | 1 | 76,076,655 | 9.86 × 10^-13^ |  | 12 | 0 | 0 |  | 3 | 26 | 38 |
| BIEC2-32520 | 1 | 76,154,827 | 1.45 × 10^-14^ |  | 13 | 0 | 0 |  | 3 | 28 | 38 |
| BIEC2-32546 | 1 | 76,229,305 | 7.39 × 10^-07^ |  | 13 | 0 | 0 |  | 16 | 36 | 17 |
| BIEC2-32706 | 1 | 76,631,203 | 3.77 × 10^-14^ |  | 12 | 0 | 1 |  | 2 | 23 | 44 |
| BIEC2-32912 | 1 | 76,963,647 | 6.13 × 10^-17^ |  | 12 | 0 | 1 |  | 0 | 22 | 47 |
| BIEC2-32970 | 1 | 77,220,026 | 2.22 × 10^-15^ |  | 11 | 1 | 1 |  | 0 | 23 | 46 |
| BIEC2-33182 | 1 | 77,433,355 | 1.53 × 10^-11^ |  | 11 | 1 | 1 |  | 3 | 25 | 41 |
| BIEC2-33183 | 1 | 77,433,370 | 2.14 × 10^-11^ |  | 11 | 1 | 1 |  | 3 | 24 | 41 |
| BIEC2-33188 | 1 | 77,435,722 | 3.68 × 10^-09^ |  | 11 | 2 | 0 |  | 6 | 30 | 33 |
| BIEC2-33805 | 1 | 78,269,707 | 7.01 × 10^-09^ |  | 9 | 4 | 0 |  | 3 | 25 | 38 |
| BIEC2-33837 | 1 | 78,303,728 | 3.16 × 10^-09^ |  | 9 | 4 | 0 |  | 3 | 26 | 40 |
| BIEC2-33900 | 1 | 78,377,914 | 1.74 × 10^-09^ |  | 10 | 3 | 0 |  | 4 | 27 | 38 |
| BIEC2-34068 | 1 | 78,621,508 | 2.41 × 10^-07^ |  | 10 | 3 | 0 |  | 7 | 26 | 35 |
| BIEC2-34406 | 1 | 78,953,808 | 5.66 × 10^-07^ |  | 10 | 2 | 1 |  | 7 | 36 | 24 |
| BIEC2-34512 | 1 | 79,052,105 | 7.28 × 10^-07^ |  | 11 | 1 | 1 |  | 10 | 37 | 22 |
| BIEC2-34608 | 1 | 79,254,756 | 6.42 × 10^-10^ |  | 9 | 3 | 1 |  | 2 | 25 | 42 |
| BIEC2-34666 | 1 | 79,305,704 | 6.42 × 10^-10^ |  | 9 | 3 | 1 |  | 2 | 25 | 42 |
| BIEC2-35047 | 1 | 80,361,702 | 3.36 × 10^-11^ |  | 10 | 2 | 1 |  | 2 | 27 | 40 |
| BIEC2-35086 | 1 | 80,409,804 | 7.45 × 10^-08^ |  | 11 | 1 | 1 |  | 8 | 41 | 20 |
| BIEC2-35298 | 1 | 80,711,571 | 3.16 × 10^-11^ |  | 9 | 3 | 1 |  | 1 | 20 | 48 |
| BIEC2-35307 | 1 | 80,724,601 | 6.14 × 10^-11^ |  | 9 | 3 | 1 |  | 1 | 20 | 46 |
| BIEC2-35396 | 1 | 80,965,484 | 2.36 × 10^-07^ |  | 9 | 3 | 1 |  | 5 | 25 | 39 |
| BIEC2-35714 | 1 | 81,858,337 | 1.92 × 10^-07^ |  | 11 | 0 | 2 |  | 9 | 33 | 27 |
| BIEC2-36540 | 1 | 84,711,902 | 1.21 × 10^-06^ |  | 7 | 5 | 1 |  | 3 | 25 | 40 |
| BIEC2-36578 | 1 | 84,873,250 | 6.70 × 10^-10^ |  | 7 | 4 | 2 |  | 0 | 22 | 47 |
| BIEC2-36598 | 1 | 84,981,382 | 9.75 × 10^-10^ |  | 9 | 2 | 2 |  | 2 | 27 | 40 |
| BIEC2-36621 | 1 | 85,025,168 | 9.75 × 10^-10^ |  | 9 | 2 | 2 |  | 2 | 27 | 40 |
| BIEC2-36625 | 1 | 85,028,493 | 9.75 × 10^-10^ |  | 9 | 2 | 2 |  | 2 | 27 | 40 |
| BIEC2-36628 | 1 | 85,030,689 | 9.75 × 10^-10^ |  | 9 | 2 | 2 |  | 2 | 27 | 40 |
| BIEC2-36679 | 1 | 85,302,089 | 1.20 × 10^-06^ |  | 10 | 2 | 1 |  | 8 | 36 | 25 |
| BIEC2-36756 | 1 | 85,624,691 | 7.25 × 10^-10^ |  | 7 | 4 | 2 |  | 0 | 23 | 46 |
| BIEC2-36918 | 1 | 86,544,396 | 7.56 × 10^-07^ |  | 5 | 7 | 1 |  | 1 | 23 | 45 |
| BIEC2-36944 | 1 | 86,656,572 | 1.96 × 10^-07^ |  | 5 | 6 | 2 |  | 0 | 27 | 42 |
| BIEC2-36978 | 1 | 86,837,051 | 2.34 × 10^-08^ |  | 7 | 4 | 2 |  | 1 | 26 | 42 |
| BIEC2-36985 | 1 | 86,848,580 | 2.34 × 10^-08^ |  | 7 | 4 | 2 |  | 1 | 26 | 42 |
| BIEC2-36996 | 1 | 86,965,305 | 2.06 × 10^-08^ |  | 7 | 4 | 2 |  | 1 | 24 | 44 |
| BIEC2-37016 | 1 | 87,076,114 | 3.37 × 10^-08^ |  | 7 | 4 | 2 |  | 1 | 28 | 39 |
| BIEC2-37017 | 1 | 87,079,561 | 3.31 × 10^-07^ |  | 7 | 4 | 2 |  | 2 | 28 | 39 |
| BIEC2-37132 | 1 | 87,641,854 | 1.97 × 10^-08^ |  | 7 | 5 | 1 |  | 1 | 34 | 34 |
| BIEC2-171284 | 12 | 6,234,389 | 8.82 × 10^-07^ |  | 5 | 8 | 0 |  | 64 | 5 | 0 |
| BIEC2-411530 | 18 | 40,953,961 | 7.53 × 10^-07^ |  | 6 | 3 | 4 |  | 1 | 22 | 46 |
| BIEC2-411567 | 18 | 41,145,097 | 7.28 × 10^-07^ |  | 6 | 3 | 4 |  | 1 | 21 | 47 |
