# Supplementary material for: A nonsense mutation in B3GALNT2 is concordant with hydrocephalus in Friesian horses
Source: BMC Genomics. 2015 Oct 9;16:761. doi: 10.1186/s12864-015-1936-z (PMC4600337; doi:10.1186/s12864-015-1936-z)
Supplement: Additional file 3: — Genes located in the ECA1 region shared in a homozygous state by hydrocephalus cases. Start and stop position (in base pair; Equus caballus EquCab2.0 reference genome [29]), symbol and description of genes located in the region of 1.47 Mb in length that is shared in a homozygous state by hydrocephalus cases. (DOCX 21 kb) [file 12864_2015_1936_MOESM3_ESM.docx]

## Genes located in the ECA1 region shared in a homozygous state by hydrocephalus cases

| Start | Stop | Symbol | Description |
| --- | --- | --- | --- |
| 74,838,844 | 74,905,562 | ACTN2 | actinin, alpha 2 |
| 74,977,743 | 75,030,762 | HEATR1 | HEAT repeat containing 1 |
| 75,030,846 | 75,049,803 | LGALS8 | lectin, galactoside-binding, soluble, 8 |
| 75,049,880 | 75,073,822 | LOC102148322 | uncharacterized LOC102148322 |
| 75,078,519 | 75,137,322 | EDARADD | EDAR-associated death domain |
| 75,227,578 | 75,282,436 | ERO1LB | ERO1-like beta (S. cerevisiae) |
| 75,228,451 | 75,337,404 | GPR137B | G protein-coupled receptor 137B |
| 75,400,339 | 75,477,555 | NID1 | nidogen 1 |
| 75,408,501 | 75,410,641 | LOC102148780 | charged multivesicular body protein 1b-2-like |
| 75,553,920 | 75,750,121 | LYST | lysosomal trafficking regulator |
| 75,751,068 | 75,751,955 | LOC100050801 | ferritin light chain-like |
| 75,785,691 | 75,833,553 | GNG4 | guanine nucleotide binding protein (G protein), gamma 4 |
| 75,859,296 | 75,909,376 | B3GALNT2 | beta-1,3-N-acetylgalactosaminyltransferase 2 |
| 75,908,936 | 75,976,451 | TBCE | tubulin folding cofactor E |
| 75,998,476 | 76,009,175 | GGPS1 | geranylgeranyl diphosphate synthase 1 |
| 76,011,143 | 76,158,986 | ARID4B | AT rich interactive domain 4B (RBP1-like) |
| 76,162,957 | 76,179,452 | RBM34 | RNA binding motif protein 34 |
| 76,182,903 | 76,198,754 | TOMM20 | translocase of outer mitochondrial membrane 20 homolog (yeast) |

Start and stop position (in base pair; *Equus caballus* EquCab2.0 reference genome [29]), symbol and description of genes located in the region of 1.47 Mb in length that is shared in a homozygous state by hydrocephalus cases.
