# Supplementary material for: A nonsense mutation in B3GALNT2 is concordant with hydrocephalus in Friesian horses
Source: BMC Genomics. 2015 Oct 9;16:761. doi: 10.1186/s12864-015-1936-z (PMC4600337; doi:10.1186/s12864-015-1936-z)
Supplement: Additional file 5: — DNA sequence variations in regions of homozygosity in Friesian horses with hydrocephalus. A table with information on DNA sequence variations in regions of homozygosity in Friesian horses with hydrocephalus. (DOCX 20 kb) [file 12864_2015_1936_MOESM5_ESM.docx]

## DNA sequence variations in regions of homozygosity in Friesian horses with hydrocephalus

Chromosome (ECA = *Equus caballus*), position (in base pair; *Equus caballus* EquCab2.0 reference genome [29]), strand orientation, nucleotide, predicted consequence on amino acid, located within gene and type of variation (snv = single nucleotide variation, indel = insertion or deletion) in the regions of homozygosity in Friesian horses with hydrocephalus. The grey shaded variations segregate with the phenotype.

| ECA | Position | Orientation | Nucleotide | Amino acid | Gene | Type |
| --- | --- | --- | --- | --- | --- | --- |
| 1 | 74,986,528 | 1 | G/C | D393H | *HEATR1* | snv |
| 1 | 74,992,783 | 1 | G/A | V538I | *HEATR1* | snv |
| 1 | 74,994,656 | 1 | G/A | E642K | *HEATR1* | snv |
| 1 | 74,996,030 | 1 | T/C | I707T | *HEATR1* | snv |
| 1 | 75,007,407 | 1 | G/A | na | *HEATR1* | snv |
| 1 | 75,020,726 | 1 | G/A | V1560I | *HEATR1* | snv |
| 1 | 75,263,816 | 1 | G/A | na | *ERO1LB* | snv |
| 1 | 75,337,324 | -1 | A/G | L29P | *GPR137B* | snv |
| 1 | 75,337,339 | -1 | T/G | N24T | *GPR137B* | snv |
| 1 | 75,400,523 | 1 | G/- | na | *NID1* | indel |
| 1 | 75,400,573 | 1 | G/- | na | *NID1* | indel |
| 1 | 75,472,312 | 1 | C/T | S1115F | *NID1* | snv |
| 1 | 75,614,207 | 1 | T/C | L884P | *LYST* | snv |
| 1 | 75,634,858 | 1 | A/G | K1715E | *LYST* | snv |
| 1 | 75,907,505 | 1 | C/T | Q475* | *B3GALNT2* | snv |
| 1 | 76,167,112 | 1 | C/A | D147E | *RBM34* | snv |
