# Supplementary material for: A nonsense mutation in B3GALNT2 is concordant with hydrocephalus in Friesian horses
Source: BMC Genomics. 2015 Oct 9;16:761. doi: 10.1186/s12864-015-1936-z (PMC4600337; doi:10.1186/s12864-015-1936-z)
Supplement: Additional file 6: — Linkage disequilibrium between the candidate mutation in B3GALNT2 and surrounding SNPs. A figure with LD between the candidate mutation in B3GALNT2 and surrounding SNPs. (DOCX 174 kb) [file 12864_2015_1936_MOESM6_ESM.docx]

### Linkage disequilibrium between the candidate mutation in *B3GALNT2* and surrounding SNPs


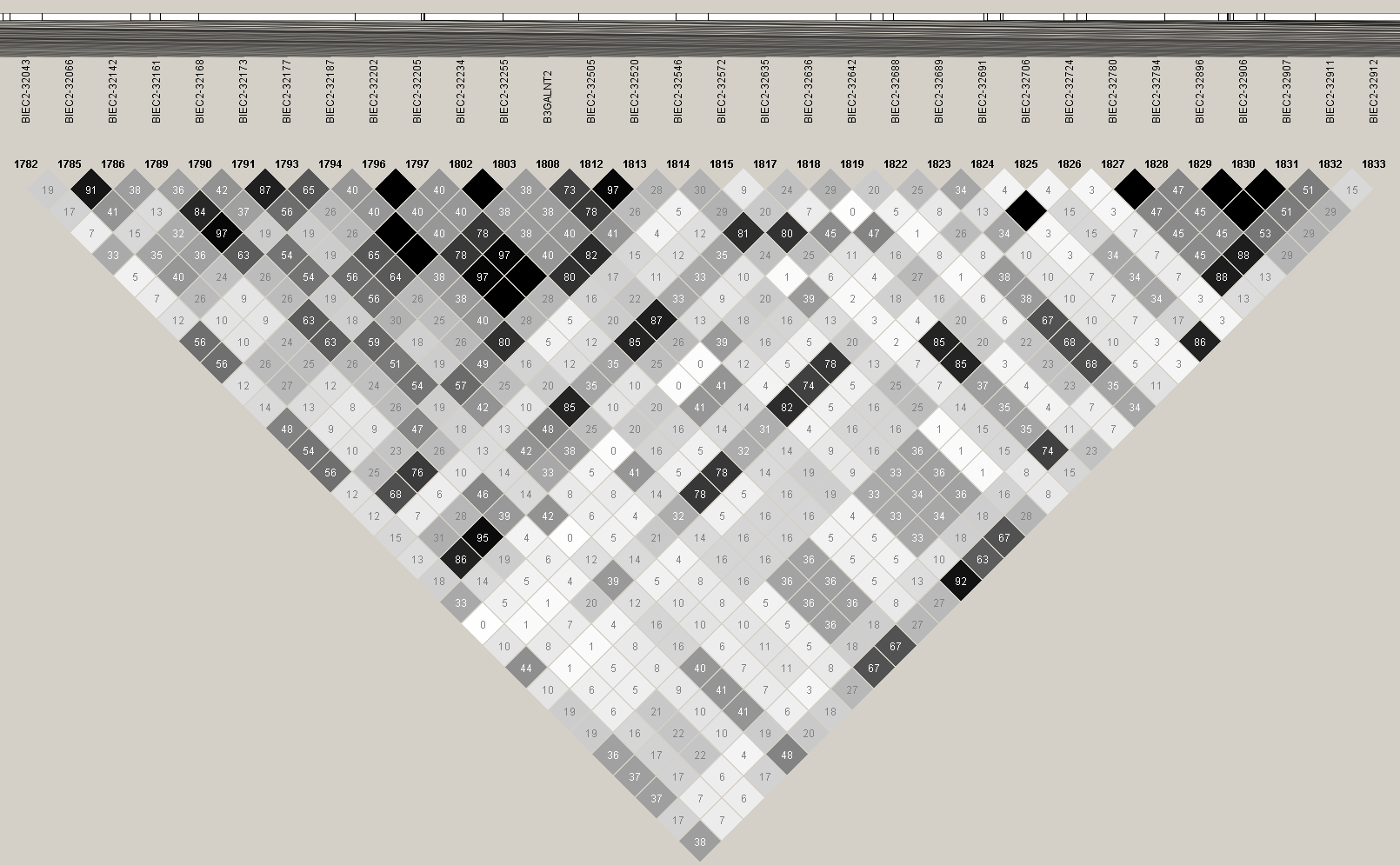
LD (pair-wise $r^{2}$) between the candidate mutation in *B3GALNT2* and surrounding SNPs based on genotypes for the mutation of 13 cases, 45 controls and 3 dams of cases and based on SNP genotypes of all 13 cases and 69 controls.

homozygous region in all 13 cases (Figure 2)

ECA1:74,938,827-76,370,694 mutation BIEC2-32912 associated (*P* = 6.13 × 10^-17^) with hydrocephalus
